# Insecticide susceptibility status of *Phlebotomus (Paraphlebotomus) sergenti *and *Phlebotomus (Phlebotomus) papatasi *in endemic foci of cutaneous leishmaniasis in Morocco

**DOI:** 10.1186/1756-3305-5-51

**Published:** 2012-03-19

**Authors:** Chafika Faraj, Souad Ouahabi, El Bachir Adlaoui, Mohammed El Elkohli, Lhousseine Lakraa, Mohammed El Rhazi, Btissam Ameur

**Affiliations:** 1 Laboratoire d'Entomologie Médicale, Institut National d'Hygiène, 27 Avenue Ibn Batouta, Agdal, Rabat 10090, Morocco; 2 Service de lutte Antivectorielle, Direction de l'Epidémiologie et de Lutte contre les Maladies, Agdal, Rabat 10080, Morocco

**Keywords:** Sandflies, Insecticide susceptibility, Lambdacyhalothrin, DDT, Malathion, *Phlebotomus sergenti*, *Phlebotomus papatasi*, Morocco

## Abstract

**Background:**

In Morocco, cutaneous leishmaniasis is transmitted by *Phlebotomus sergenti *and *Ph. papatasi*. Vector control is mainly based on environmental management but indoor residual spraying with synthetic pyrethroids is applied in many foci of *Leishmania tropica*. However, the levels and distribution of sandfly susceptibility to insecticides currently used has not been studied yet. Hence, this study was undertaken to establish the susceptibility status of *Ph. sergenti *and *Ph. papatasi *to lambdacyhalothrin, DDT and malathion.

**Methods:**

The insecticide susceptibility status of *Ph. sergenti *and *Ph. papatasi *was assessed during 2011, following the standard WHO technique based on discriminating dosage. A series of twenty-five susceptibility tests were carried out on wild populations of *Ph. sergenti *and *Ph. papatasi *collected by CDC light traps from seven villages in six different provinces. Knockdown rates (KDT) were noted at 5 min intervals during the exposure to DDT and to lambdacyhalothrin. After one hour of exposure, sandflies were transferred to the observation tubes for 24 hours. After this period, mortality rate was calculated. Data were analyzed by Probit analysis program to determine the knockdown time 50% and 90% (KDT50 and KDT90) values.

**Results:**

Study results showed that *Ph.sergenti *and *Ph. papatasi *were susceptible to all insecticides tested. Comparison of KDT values showed a clear difference between the insecticide knockdown effect in studied villages. This effect was lower in areas subject to high selective public health insecticide pressure in the framework of malaria or leishmaniasis control.

**Conclusion:**

*Phlebotomus sergenti *and *Ph. papatasi *are susceptible to the insecticides tested in the seven studied villages but they showed a low knockdown effect in Azilal, Chichaoua and Settat. Therefore, a study of insecticide susceptibility of these vectors in other foci of leishmaniasis is recommended and the level of their susceptibility should be regularly monitored.

## Background

Both cutaneous (anthroponotic and zoonotic) and visceral leishmaniasis (VL) are present in Morocco. Cutaneous leishmaniasis (CL) is caused by *Leishmania major* Yakimoff and Schokhor, *L. tropica* Wright, or *L. infantum* Nicolle, while VL is caused by *L. infantum *[1]. These diseases are considered to be a serious public health concern for Morocco. Until 1999, it was mainly limited to rural areas with an hypoendemic transmission [2]. In 2001, the Moroccan Ministry of Health (MMOH) reported 2019 CL cases caused by *L. major *and *L. tropica *[3]. Since then, the disease has spread gradually from the south to the north and from rural to sub urban regions. In 2010 MMOH reported 2263 cases caused by *L. tropica *and 6444 cases caused by *L. major *[4].

Among sandfly species involved in leishmania transmission in Morocco, *Phlebotomus (Paraphlebotomus) sergenti *Parrot and *Ph.(Phlebotomus) papatasi *Scopoli are the main vectors of Anthroponotic CL and Zoonotic CL respectively [5,6]. These species show large anthropophilic behaviour in peri-domestic and domestic habitats and they are widespread throughout the country in both rural and urban areas [7].

Until 2000, control measures against leishmaniasis were based only on treatment of human cases with antimonial drugs and on rodent control for ZCL [2]. Currently, they rely also on vector control measures. Indoor Residual Spraying (IRS), with synthetic pyrethroids, are applied in many Moroccan foci of *L. tropica*. However, these control strategies seem not to be effective to control CL throughout the country. The incidence is increasing continuously and new foci are emerging. Moreover, evaluation of chemical sandfly control points out, in some areas, the low efficiency in reducing the density of sandflies. This might be due to various factors including resistance of local sandfly populations to the insecticide in use. The pressure of insecticides used by the health sector, as well as in agricultural activities and domestic hygiene, may contribute to developing resistance in vector populations. Unfortunately, the levels and distribution of sandfly susceptibility to insecticides has not been studied in Morocco. Thus, to improve control measures against CL vectors and to provide a rational framework for choosing the suited insecticide, this study has been undertaken. It aimed to investigate the insecticide susceptibility of two Moroccan CL vectors, *Ph. sergenti *and *Ph. Papatasi*, to insecticides used in endemic areas.

## Methods

### Study area

**Figure 1:**
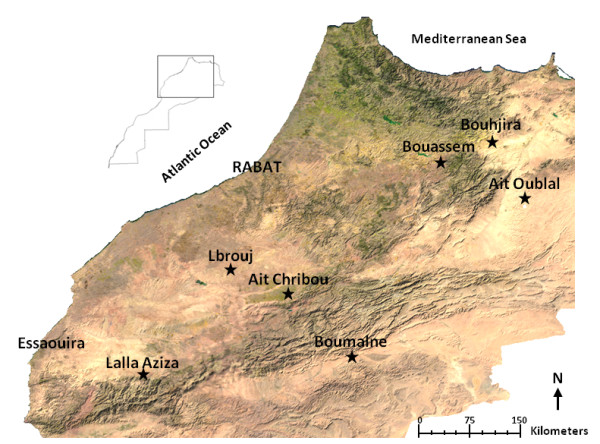
**Map showing the study sites**. ⋆ Study site.

This study was conducted in seven villages from six provinces in Morocco: Bouhjira (Taza) in the north-east, Lbrouj (Settat) in the center, Ait Chribou (Azilal) and Lalla Aziza (Chichaoua) on the High Atlas chain in the center-south of Morocco, Boumalne (Tinghir) in the south of the high Atlas Mountains, Bouassem (Boulemane) in the north west of the middle Atlas mountain and Ait Oublal (Boulemane) in the east of Morocco (Figure 1). These districts were selected as they are endemic for CL and have been subjected to different insecticide selection pressures.

### Sandfly collection

Sandflies from the regions described were collected using CDC Light Traps during the period June-October 2011. In each village, five traps were installed in different animal's shelters from sunset to sunrise. After one hour of observation, living sandflies were selected for testing. Detailed dates of tests are given in Tables 1 and 2.

**Table 1:** Insecticide susceptibility tests of wild collected *Phlebotomus sergenti*

Province	locality	Coor- donates	Month	Insecticide	Nbre exposed	Mortality (%)	KDT50 mn (CI)	KDT90 mn (CI)	Observed KDT100 mn
Azilal	Ait chribou	6°20' W 32°11'N		Lambdacyhalothrin	56	100	29.3 (27.2-31.4)	64.7 (57.9-74.6)	> 60
			
			October	DDT	52	100	33.8 (31.5-36.2)	71.9 (63.8-84.2)	> 60
			
				Malathion	51	100	-	-	-

Boulmane	Bouassam	4°33' W 33°31' N		Lambdacyhalothrin	39	100	12.6 (8.4-16.4)	25.6 (19.5-43.9)	30
			
			September	DDT	51	100	11.0 (8.9-12.9)	20.6 (17.4-26.6)	25

Chichaoua	Lalla aziza	8°44' W 31°03' N		Lambdacyhalothrin	47	100	25.5 (23.0-28.1)	72.5 (62.0-89.4)	> 60
			
			June	DDT	40	100	32.0 (28.8-35.7)	95.7 (77.7-129.0)	> 60

Settat	Lbrouj	07°36' W 32°29' N		Lambdacyhalothrin	44	100	26.8 (24.2-29.5)	73.0 (62.4-90.3)	> 60
			
			July	DDT	54	100	30.7 (28.2-33.5)	83.8 (71.6-103.5)	> 60

Taza	Bouhajra	04° 01' W 34°13' N		Lambdacyhalothrin	43	100	15.6 (12.6-18.3)	26.3 (22.0-36.1)	30
			
			September	DDT	41	100	11.8 (10.5-13.1)	21.5 (19.2-24.8)	25

Tinghir	Boumalne	6°00' W 31°18' N		Lambdacyhalothrin	45	100	14.3 (11.1-17.2)	27.9 (23.1-36.7)	30
			
			July	DDT	41	100	12.7 (11.2-14.1)	26.2 (23.6-29.9)	35
			
				Malathion	46	100	-	-	-

KDT50/KDT90: Knock down time for 50% and 90% of exposed sandflies with confidence intervals (CI) at 5% level

**Table 2:** Insecticide susceptibility tests of wild collected *Phlebotomus papatasi*

Province	locality	Coor- donates	Month	Insecticide	Nbre exposed	Mortality (%)	KDT50 mn (CI)	KDT90 mn (CI)	Observed KDT100 mn
Boulmane	Ait oublal	2° 27' W 32° 32' N		Lambdacyhalothrine	54	100	11.9 (48.1-16.4)	25.6 (19.6-35.8)	30
			
			September	DDT	59	100	22.4 (18.6-25.8)	43.5 (36.6-57.3)	45
			
				Malathion	58	100	-	-	-

Chichaoua	Lalla aziza	8°44' W 31°03' N	June	Lambdacyhalothrin	42	100	32.4 (29.1-36.3)	102.1 (82.0-139.7)	> 60

Settat	lbrouj	07°36' W 32°29' N		Lambdacyhalothrine	47	100	33.3 (30.9-35.9)	71.3 62.9-84.3)	> 60
			
			July	DDT	49	100	32.9 (30.6-35.4)	71.0 (62.8-83.7)	> 60

Taza	Bouhajra	04° 01' W 34°13' N		Lambdacyhalothrin	30	100	14.5 (13.0-15.9)	23.4 (21.0-27.3)	25
			
			September	DDT	35	100	15.3 (12.0-18.3)	27.3 (22.4-38.7)	35

Tinghir	Boumalne	6°00' W 31°18' N		Lambdacyhalothrin	51	100	16.4 (12.1-20.4)	38.8 (30.2-60.3)	40
			
			July	DDT	48	100	18.0 (13.5-22.2)	42.3 (33.0-65.9)	45
			
				Malathion	31	100	-	-	-

KDT50/KDT90: Knock down time for 50% and 90% of exposed sandflies with confidence intervals (CI) at 5% level

### Bioassay tests

Sandflies were exposed to three different insecticides. In each province and depending on sandfly density, tests were carried out, by priority, to lambdacyhalothrin 0.05%, DDT 4% then Malathion 5%.

Insecticide susceptibility tests were carried out following WHO standard procedures using discriminating dosage [8], where field populations were exposed to a concentration of insecticide defined as diagnostic. DDT discriminating dosage, established by WHO, for the genus *Phlebotomus *is 4/1 (exposition to 4% DDT impregnated paper for 1 hour) [9]. However, no standardized discriminating concentrations or time of exposure to lambdacyhalothrin and malathion have been given for sandflies by WHO as is the case for malaria vectors. We decided, hence, based on a literature review to consider one hour exposure to 0.05% lambdacyhalothrin and 5% malathion as a discriminating concentration [10-12].

Standard WHO testing procedures were applied to assess the insecticide resistance/susceptibility using the test-kit tubes [7]. In each test, three replicates of about 25 sandflies (not yet identified), according to the availability of the sandflies, were performed. For each batch a control test was performed using the corresponding control papers. Knockdown rates were noted at 5 min intervals during the insecticide exposure to DDT and lambdacyhalothrin. After one hour of exposure, sandflies were transferred to the observation tube and kept in appropriate conditions (25 ± 2°C and 80% ± 10% relative humidity) for 24 hours. Sufficient relative humidity was ensured by putting small pieces of cotton wool impregnated with distilled water on the top of the cups. After 24 hours of observation, alive and dead sandflies per cup were identified [13] and counted. Mortality rates were calculated for each target species

### Data analysis

Data analysis was made using log-probit analysis software (WinDL version 2.0) developed by CIRAD-CA/MABIS [14]. It allows calculation of KDT50, KDT90 (Time involving respectively the knockdown of 50% and 90% of tested sandflies) and their confidence intervals.

## Results

Twenty-five sets of susceptibility tests (11 for lambdacyhalothrin, 10 for DDT and 4 for malathion) of *Ph. sergenti *and *Ph. papatasi *in seven villages were carried out. Results of bioassays are presented by village in Tables 1 and 2. *Ph. sergenti *and *Ph. papatasi *field populations tested were susceptible to lambdacyhalothrin, DDT and malathion. No specimen survived after 60 min exposure to these insecticides.

Nevertheless, considering the KDT values observed, the results show a difference in response among populations of both *Ph. sergenti *and *Ph. papatasi *to lambdacyhalothrin and DDT. Generally, KDT values for DDT were higher when compared with those for lambdacyalothrin. In the villages of Tinghir, Boulmane and Taza, lambdacyhalothrin and DDT induced a *Ph. sergenti *knockdown of 100% after less than 30 min of exposure. Whilst in those of Azilal, Settat and Chichaoua, the average sandfly knockdown rate at 1 hour varied between 90 and 93%. Similarly, for *Ph. papatasi*, lambdacyhalothrin and DDT provided 100% knockdown after less than 45 min in the villages of Boulmane, Taza and Tinghir even as, in those of Chichaoua and Settat knockdown rates varied between 85 and 93% following one hour.

## Discussion

The present paper reports the results of the first study on the insecticide susceptibility of *Ph. sergenti *and *Ph. papatasi *in Morocco.

Several studies have investigated the susceptibility of sandflies to insecticides around the world. However, the methods used in those studies were not identical i.e. insecticide concentration and time of exposure varied. Most tests have been performed on reared sandfly colonies using dose-mortality bioassays [10,11,15], or Time-mortality bioassays [12,16-18]. However, there are few studies that have focused on sandflies collected in the field and adopted the discriminating concentration [19-21]. We decided to carry out this study by using diagnostic dose bioassays since this method is easy, fast and requires only a small number of specimens compared to dose-mortality or time-mortality bioassays. It is, thus, more convenient for testing the susceptibility of field populations of sandflies, considering their limited density in the field.

Our results indicate that the leishmaniasis vectors *Ph. sergenti *and *Ph. papatasi *collected in this study are susceptible to lambdacyhalothrin, DDT and malathion.

These results are in line with those of several studies carried out in other countries and which concluded that, generally, sandflies are still susceptible to the majority of insecticides in use. Indeed, studies in Italy have found no evidence of DDT, pyrethroid or organophosphate resistance in *Ph. perniciosus, Ph. perfiliewi *or *Ph. papatasi *[18,22]. Aboul Ela *et al*. [10] and Fahmy *et al*. [17] reported the susceptibility of egyptian field populations of *Ph. papatasi *to DDT, dieldrine, malathion, propoxur, permethrin and deltamethrin. Further studies in Egypt confirmed the susceptibility of *Ph. langeroni, Ph. papatasi *and *Ph. sergenti *to six insecticides (DDT, resmethrin, cyfluthrin, permethrin, bendiocarb and malathion) [11]. In Israel, susceptibility of *Ph. papatasi *to DDT and permethrin was reported [23]. Moreover, studies in Venezuela revealed no indication of resistance in *Lutzomyia longipalpis *to propoxur, malathion, deltamethrin or lambdacyhalothrin [24]. But recently, Alexander *et al*. [12] point out a significantly reduced susceptibility in this species to malathion, fenitrothion, lambdacyhalothrin, permethrin and deltamethrin in Brazil.

The reports on insecticide resistance in phlebotomine sandflies are few. To date, the only indicated DDT resistance has been reported in India in *Ph. papatasi *[19,21,25-27] and *Ph. argentipes *[20,27-29]. These species were, then, found to be resistant to pyrethroids [27]. It is to be noticed that a tolerance of *Ph. papatasi *to DDT was signalled in Iran [16,30]. Considering *Ph. sergenti*, there have been no records of insecticide resistance until now.

In view of the KDT values observed, results of this study revealed that sandfly populations of Boulmane, Tinghir and Taza were more sensitive to lambdacyhalothrin and DDT compared with those of Azilal, Chichaoua and Settat. In the first group, either for *Ph. sergenti *and *Ph. papatasi*, KDT100 were less than 30 min. Whereas in the second group, they were over 60 min, Martinez-Torres *et al*. [31] estimate that the decline in the knockdown effect can be considered as an early indicator of resistance development as it can be important before the observation of mortality reduction. This can be observed in the WHO test only when the sandfly population consists of a large proportion of homozygosity of a resistance gene. Chandre *et al*. [32] obtained similar results in susceptible strains of the malaria vector *Anopheles gambiae*, homozygous and heterozygous for the resistance gene. The observed decreased knockdown effect in the sandfly populations of Azilal, Chichaoua and Settat provinces probably resulted from DDT or pyrethroid IRS to control malaria or leishmaniasis. In Azilal, IRS with DDT to control malaria was stopped in the early 1990's. In 2010, IRS was essentially based on the use of pyrethroids (mainly alphacypermethrin) to control leishmaniasis, but only in a few villages with high incidence. Nevertheless, in Chichaoua, an insecticide spraying program to control leishmaniasis was started in 2000 and continued up till now. In Settat, the last malaria case was reported in 1995. Last IRS using DDT were carried out before this date to interrupt malaria transmission. The first outbreak of leishmaniasis was in 2007 and IRS with lambdacyhalothrin were then conducted to control transmission in this focus. However, no insecticide use in public health control programs have been reported during the last 30 years in Boulmane and Taza. In Tinghir, households have never been treated with DDT. IRS with pyrethroids to control sandflies were carried out since 2010 with alphacypermrthrin. This emphasizes that reduced knockdown in *Ph. sergenti *and *Ph. papatasi *is principally attributed to indoor insecticide spraying and their frequency of use.

## Conclusion

*Phlebotomus sergenti and Ph. papatasi *are still susceptible to the insecticides tested in the six studied provinces in Morocco but they showed a low Knockdown effect in Azilal, Chichaoua and Settat. Thus, a study of insecticide susceptibility of these vectors in other leishmaniasis foci is suggested and the spectrum of this susceptibility should be regularly followed up.

## Competing interests

The authors declare that they have no competing interests.

## Authors' contributions

CF conceived and designed the study and drafted the manuscript. SO Carried out sandflies identification and participated in the review of the manuscript, EA carried out the data analysis and participated in the review of the manuscript. ME, LL, ME carried out the field work and the bioassay tests. BA has given financial support and participated in the review of the manuscript. All authors read and approved the final version of the manuscript.
